# Physiological and Molecular Mechanism of Nitric Oxide (NO) Involved in Bermudagrass Response to Cold Stress

**DOI:** 10.1371/journal.pone.0132991

**Published:** 2015-07-15

**Authors:** Jibiao Fan, Ke Chen, Erick Amombo, Zhengrong Hu, Liang Chen, Jinmin Fu

**Affiliations:** 1 Key Laboratory of Plant Germplasm Enhancement and Specialty Agriculture, Wuhan Botanical Garden, Chinese Academy of Sciences, Wuhan, Hubei 430074, China; 2 University of Chinese Academy of Sciences, 19 Yuquan Road, Beijing 100049, China; University of Vigo, SPAIN

## Abstract

Bermudagrass is widely utilized in parks, lawns, and golf courses. However, cold is a key factor limiting resource use in bermudagrass. Therefore, it is meaningful to study the mechanism of bermudagrass response to cold. Nitric oxide (NO) is a crucial signal molecule with multiple biological functions. Thus, the objective of this study was to investigate whether NO play roles in bermudagrass response to cold. Sodium nitroprusside (SNP) was used as NO donor, while 2-phenyl-4,4,5,5-tetramentylimidazoline-l-oxyl-3-xide (PTIO) plus *N^G^*-nitro-L-arginine methyl ester (L-NAME) were applied as NO inhibitor. Wild bermudagrass was subjected to 4 °C in a growth chamber under different treatments (Control, SNP, PTIO + L-NAME). The results indicated lower levels of malondialdehyde (MDA) content and electrolyte leakage (EL), higher value for chlorophyll content, superoxide dismutase (SOD) and peroxidase (POD) activities after SNP treatment than that of PTIO plus L-NAME treatments under cold stress. Analysis of Chlorophyll (Chl) *a* fluorescence transient displayed that the OJIP transient curve was higher after treatment with SNP than that of treated with PTIO plus L-NAME under cold stress. The values of photosynthetic fluorescence parameters were higher after treatment with SNP than that of treated with PTIO plus L-NAME under cold stress. Expression of cold-responsive genes was altered under cold stress after treated with SNP or PTIO plus L-NAME. In summary, our findings indicated that, as an important strategy to protect bermudagrass against cold stress, NO could maintain the stability of cell membrane, up-regulate the antioxidant enzymes activities, recover process of photosystem II (PSII) and induce the expression of cold-responsive genes.

## Introduction

Low temperature is a critical environmental factor that influences geographical distribution of botanical species [1]. Plant growth and development were severely impacted by cold stress; simultaneously crop yield was severely limited under low temperature [2]. Exposure of plants to cold stress often alters the expression of numerous genes, resulting in changes in molecular, cellular and physiological metabolism processes [3]. The best understood pathway of plant response to cold is ICE1-CBF-COR cascade. When plants were exposed to cold, expression of *ICE1*, *CBF* and *COR* genes were up-regulated [4].As for physiological and metabolic changes under cold condition, photosynthetic carbon metabolism was found to be interrupted in *Arabidopsis* [5]. Additionally, MDA content and EL values increased under cold stress [6,7] suggesting significant oxidative damage. It was reported that soluble sugars and proline accumulated during cold acclimation [8]. Activities of antioxidase were also altered after cold stress treatment, precisely, SOD and APX activities were elevated under cold stress in Scots pine (*Pinus sylvestris* L.) [9]. POD activity increased after low temperature treatment in rice (*Oryza sativa* L.) [10].

Nitric oxide (NO) was reported to be a vital signal molecule that is necessary for multiple biological functions in plants [11,12]. NO is involved in seed germination, evoked de-etiolation and diminished elongation of hypocotyls [13], consequently regulating growth, development, maturation and senescence of plants [14]. NO was also observed to be essential in plant stress resistance. Santa-Cruz reported that NO could protect soybean plants against ultraviolet-B radiation [15]. NO was also found to be positively involved in heat and salt stresses [16]. Besides, it was reported that NO was a key factor in conferring plant disease resistance [17]. It was documented that NO could alleviate cold injury of Japanese plums (*Prunus salicina* Lindell) [18].

As a most important physiological process in plants, photosynthesis is sensitive to abiotic stress, and the primary target for the stress such as chilling is at the reaction center of PSII [19]. It was also showed that the binding sites of NO in the PSII complexes were the nonheme iron that between acceptors of primary (Q_A_) and secondary (Q_B_) quinine [20]. Therefore, it is meaningful to study the function of NO in photosynthetic system under abiotic stress. Chlorophyll *a* fluorescence (OJIP) is widely used as a fast and simple tool in studies to measure the photosynthetic processes efficiency and kinetics that involving in PSII [21]. It is based on the principle that the fluorescence yield was modulated with the redox state of the primary quinine electron acceptor of PSII which known as Q_A_. Changes of chlorophyll *a* fluorescence index was detected under drought and chilling stress in higher plant [22,23].

Bermudagrass [*Cynodon dactylon* (L). Pers.] is widely utilized in turf system, parks, home lawns, sports fields and golf courses. As a typical warm-season grass, bermudagrass utilization is severely limited by temperature. In middle and upper latitude, it undergoes chlorosis and even withering in late autumn and winter. Cold is considered as a key factor limiting its widespread use in bermudagrass. Therefore it is critical to increase the cold tolerance of bermudagrass. As described above, NO has potential in improving the stress tolerance. Thus, the objectives of the present study were to investigate whether and how NO participate in protecting bermudagrass against cold stress.

## Materials and Methods

### Plant materials and growth conditions

Bermudagrass (*Cynodon dactylon*) was collected from wild field of Longyan city, Fujian province, China (N 24°46.558, E 116°47.066). For this was public field in China, no specific permissions were required for this location, and the field did not involve endangered or protected species. Stolons were planted in plastic pots (10 cm tall and 8 cm in diameter) filled with matrix (brown coal soil: silver sand = 1:1). To avoid the excess water and ensure soil aeration, drainage holes were drilled at the bottom of each pot. The pot-plant systems were kept in a greenhouse under conditions of light/dark 12/12 h cycle, and day/night temperature with 30/25°C.

For grass establishment, plants were grown in the pots for 2 weeks. During the period, plants were watered with full strength Hoagland nutrient solution sufficiently, until the solution drained out from the drainage holes at the bottom of the pots.

### Treatments

The established grass was transferred into growth chamber (LSC-339CF, Xingxing Group Co.,Ltd, Zhejiang, China) treated with 4°C with a 12 h photoperiod for three days. Light intensity on a horizontal plane above the canopy averaged 240 μmol·m^–2^·s^–1^ photosynthetically active radiation (PAR). Simultaneously, plants that were cultured in 30°C chamber were set as control of temperature. 100 μM SNP was used as NO donor, 200 μM PTIO and 200 μM L-NAME were used for NO inhibitors, double distilled water was used for control. Fully extended leaf samples were collected after 3 days. Harvested leaves were put into liquid nitrogen immediately, and stored at -80°C in a refrigerator f physiological analysis.

### Measurements

To extract crude enzyme, 0.2 g of fresh leaves were ground into fine powder in liquid nitrogen. The powder was mixed with 4 mL sodium phosphate buffer (150 mM, pH 7.0) which was pre-cooled at 4°C, the homogenate was transferred into 10 mL centrifuge tube, and centrifuged for 20 min with 13000 g at 4°C. The supernatant was the crude enzyme solution to be determined.

MDA content was measured by thiobarbituric acid (TBA) method according to Hu et al. (2012) [24] with slight modification. 1 mL of crude enzyme solution was added into 2 mL MDA reaction buffer that consisted of 0.5% (v/v) TBA and 20% (v/v) trichloroacetic acid. The mixture was treated in a water bath at 95°C for 30 min, cooled to room temperature and centrifuged at 13000 g at 20°C for 10 min. The supernatant was recorded for absorbance at 532 nm and 600 nm with a spectrophotometer. MDA content was calculated with following formula:

$$\text{MDA }\left(\text{mol }g^{-1}\text{ FW}\right)=\left[\left(\text{OD}532-\text{OD}600\right)\;\times \text{ L}\right]\;/\;\left(1\times \text{ε }\times \text{FW}\right)$$

Where L indicates the volume of the extract solution, l indicates thickness of the cuvettes, ε represents the molar absorption coefficient of 155 mM^-1^ cm^-1^, and FW is the fresh weight of the leaf tissue.

To determine injury situation of cell membrane, relative EL was measured according to Hu et al. (2012) [24] with some modifications. The 0.1 g completely expanded leaves were washed 3 times with deionized water. The washed leaves were cut into about 0.5 cm fragments and transferred into 50 mL centrifuge tube filled with 15 mL deionized water, then the fragments were shaken for 24 h at room temperature. The initial conductivity of ionic solution was measured with a conductance meter (JENCO). To release the electrolytes completely, leaf tissue in the solution was autoclaved at 121°C for 10 min. the final conductivity was determined after cooling the solution at room temperature. The ratio of the two conductivity values was regarded as the relative EL.

Leaf chlorophyll content was determined by the method described by Hiscox and Israelstam (1979) [25] with slight modification. In general, 0.1 g of leaves was submerged into 10 mL dimethylsulfoxide that contained in 15 mL centrifuge tubes. Then the tubes were kept in the dark for 48 h. absorbance at 645 nm and 663 nm of the extract liquor were measured with a spectrophotometer. Chlorophyll content was calculated with the following formula:

$$\text{Chl}-\text{content}(\text{mg}\cdot L^{-1})=20.2\times \text{OD}645+8.02\times \text{OD}663$$

OD645 and OD663 indicate the absorbance of the extract liquor at 645 nm and 663 nm, respectively.

SOD activity was measured according to Hu et al. (2012) [24] with some modification. 1 mL of crude enzyme solution was added into 3 mL mixture which consisted of 2.2 mL 50mM sodium phosphate buffer (pH 7.8), 0.012 μM riboflavin, 0.039 mM methionine, 0.3 nM ethylene diaminetetraacetic acid (EDTA), 0.225 μM nitro blue tetrazolium (NBT). 3 mL reaction with no crude enzyme solution was regarded as control. The mixture was illuminated under 4000 lx fluorescent lamp for 60 min for chromogenic reaction. The absorbance at 560 nm was determined with a spectrophotometer. One unit of SOD activity was defined as amount of SOD required to inhibit NBT reduction by 50%.

POD activity was measured by the method described by Hu et al. (2012) [24]. 50 μL crude enzyme solution was mixed with 2.95 mL reaction solution constituted with 1.85 mL sodium acetate-acetic acid buffer (pH 5.0), 0.25 mL guaiacol (guaiacol was dissolved in 50% ethanol solution), and 0.075 mL H_2_O_2_. Absorbance increase at 460 nm was recorded for 3 min. Increment of 1 unit of the absorbance per minute was regarded as one unit POD activity.

Chlorophyll fluorescence was measured with a pulse-amplitude modulation (PAM) portable chlorophyll fluorimeter PAM-2500. Before measurement, plants were dark adapted for 25 min. To ensure closure of all PSII reaction centers and estimate the maximum fluorescence yield (F_m_), the OJIP transients were detected by a measuring light of 3000 μmol photons m^-2^ s^-1^. The Chl *a* fluorescence emission induced by the strong light pulses was measured and digitized between 10 μs and 320 ms. The OJIP curve was analyzed using the JIP-test.

For the gene expression analysis, total RNA was extracted with Trizol reagent (Invitrogen, Carlsbad, CA). The cDNA was synthesized with M-MLV reverse transcriptase (Promega, Madison, WI) with an oligo(dT) primer, then subjected to template for reverse transcription-polymerase chain reaction (RT-PCR) analysis. Gene specific primers were used in real-time quantitative reverse transcriptase RT-PCR (Table 1), and fluorescent dye SYBR Green (Toyobo, Osaka, Japan) was applied in detection system. According to the manual, the Real-time PCR Master Mix was used to perform Real-time PCR reaction. *Actin* gene was applied as an internal reference to normalize the data. The comparative Ct method was used to perform relative quantity of the target gene expression level [26].

**Table 1:** Primers used for expression of genes.

Gene name		Primer Sequences (5’-3’)
*POD*	F	AGGCAGCGGGGCTGAAGAAGG
	R	CCCTGACGAAGCAGTCGTGGAA
*SOD*	F	TGGGAAACATTGTTGCCAACA
	R	GCCAACAACACCACATGCCA
*CAT*	F	ATGGATCCTACCAAGTTCCGC
	R	TGCACTCGAAGAAGCCCTTGG
*LEA*	F	TCATCCCCAGCGTGTTCATCA
	R	GAGGCCGCCAAACAGAAGACA
*CBF*	F	CGTCTCCCGGAACTTGGTCC
	R	TCCAAGATGTGCCCGATCAAG

F and R represent forward and reverse, respectively.

## Results

### Effects of SNP and L-NAME plus PTIO on cell membrane stability of bermudagrass

**Fig 1:**
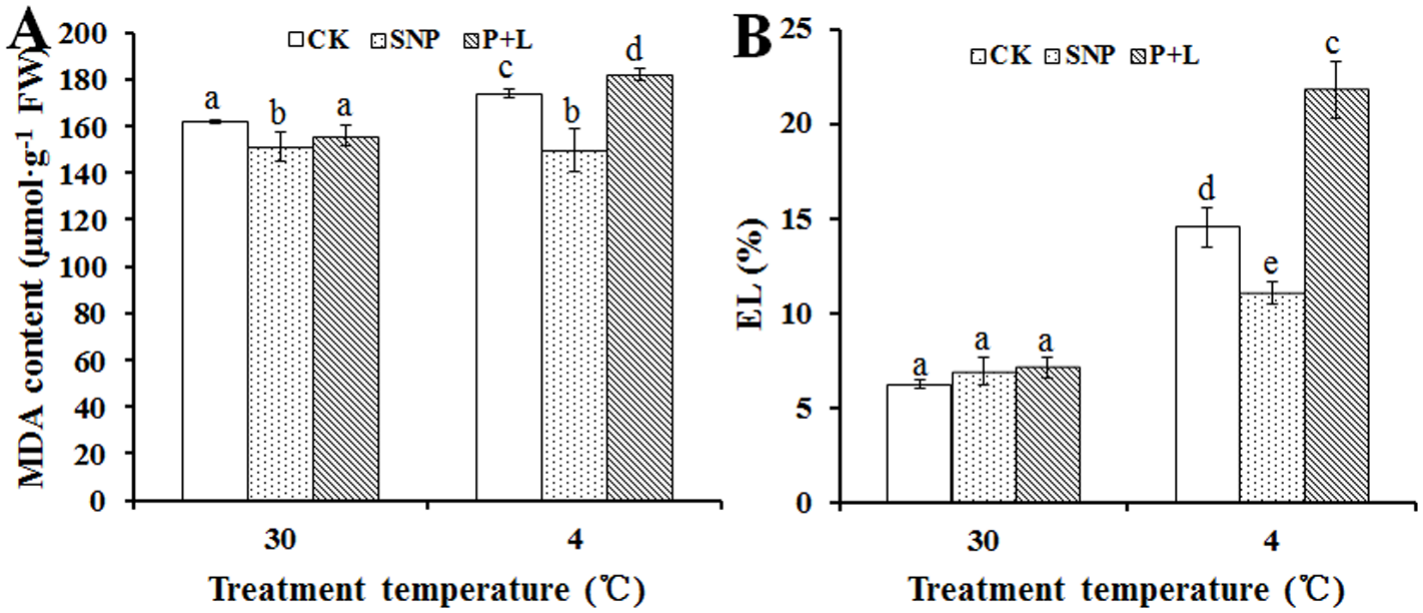
Alteration of cell membrane stability and lipid peroxidation in the leaf of bermudagrass after treated with nitric oxide (NO) donor and inhibitor under cold stress. (A) malonaldehyde (MDA) content; (B) electrolyte leakage (EL). Experiments were repeated for three times, and means were average values of MDA and EL content, respectively. Independent-samples *t* test was used to determine statistical differences. *Bars* show standard deviation. Different letters indicate statistical difference significance at *P* < 0.05 among the treatments. CK was control that treated with sterilized water, SNP was plant treated with SNP, P+L was plant treated with PTIO and L-NAME. FW was fresh weight.

MDA and EL were regarded as indicators of membrane stability. Plants with improved abiotic stress tolerance often showed higher cell membrane stability. To investigate whether NO plays a role in maintaining cell membrane stability, we analyzed the MDA and EL changes under cold stress with SNP or L-NAME plus PTIO treatments. The results showed that, under chilling stress, MDA content of the plant treated with SNP was 13.8%, lower than that of control (173.8 μmol/g). Conversely, after inhibition of NO accumulation in bermudagrass with the NOS inhibitor L-NAME plus NO scavenger PTIO, MDA content was 4.7% higher than that of control (Fig 1A). These observations indicated that lipid peroxidation was a consequence of exogenous NO, and similar results were obtained for the relative EL. The relative EL of plants treated with exogenous SNP was 3.48% lower than that of control (14.54%), and 7.23% higher than that of control in plants that NO was inhibited (Fig 1B). The results implied that NO was participating in maintaining stability of cell membrane.

### Effects of SNP and L-NAME plus PTIO on chlorophyll content of bermudagrass

**Fig 2:**
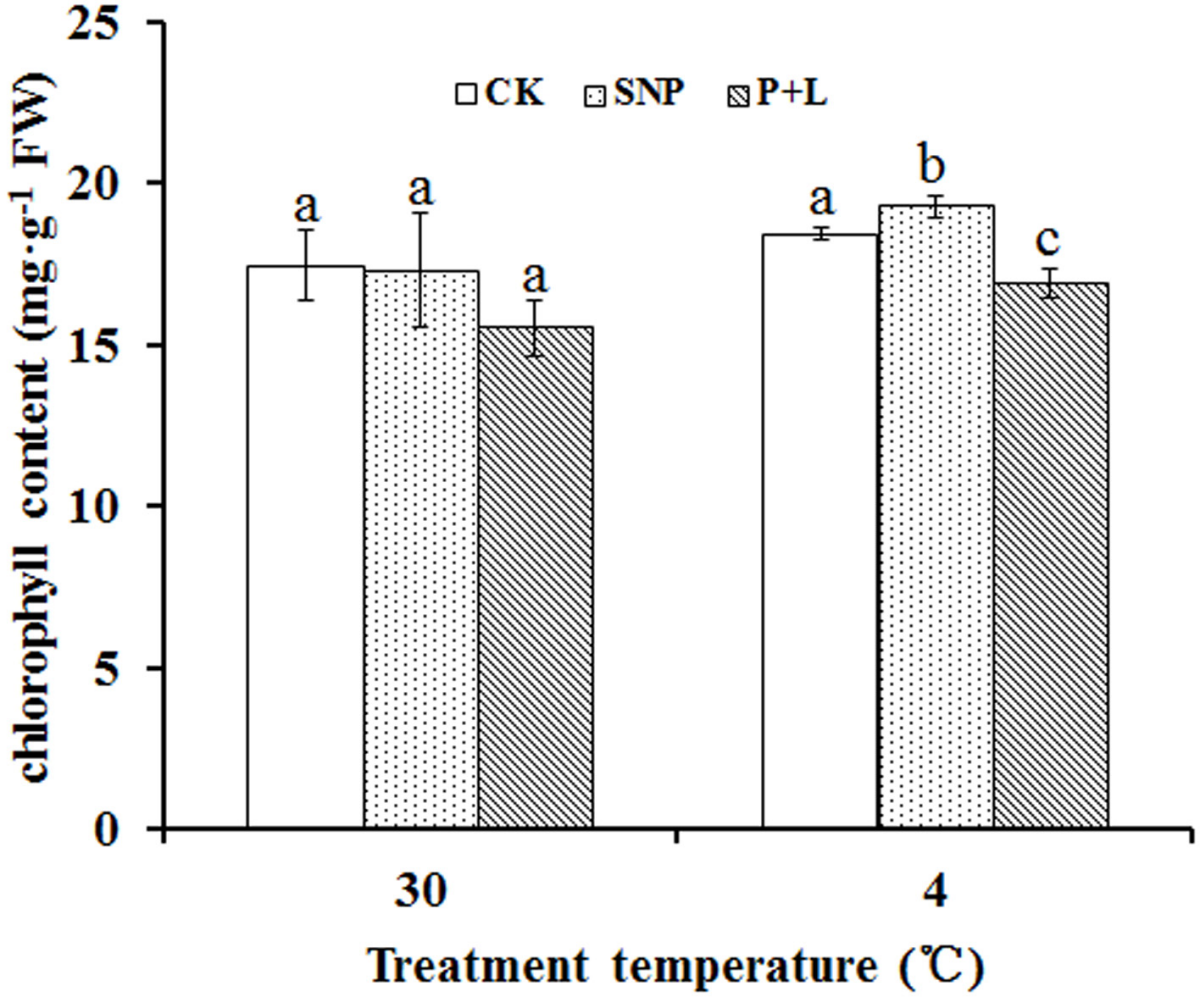
Alteration of chlorophyll content of bermudagrass after treated with nitric oxide (NO) donor and inhibitor under cold stress. Experiments were repeated for three times, and means were average values of chlorophyll content. Independent-samples *t* test was used to determine statistical differences. *Bars* show standard deviation. Different letters indicate statistical difference significance at *P* < 0.05 among the treatments. CK was control that treated with sterilized water, SNP was plant treated with SNP, P+L was plant treated with PTIO and L-NAME. FW was fresh weight.

Leaves would undergo chlorosis when plants were exposed to abiotic stress. Therefore the effect of the stress could be indicated by chlorophyll content. Under chilling stress, chlorophyll content in the plants that treated with SNP was 4.6% higher than that of control (18.43 mg/g). As expected, chlorophyll content in the plants treated with L-NAME plus PTIO was 8.3% lower than that of control (Fig 2).

### Effects of SNP and L-NAME plus PTIO on antioxidant enzyme activity of bermudagrass

**Fig 3:**
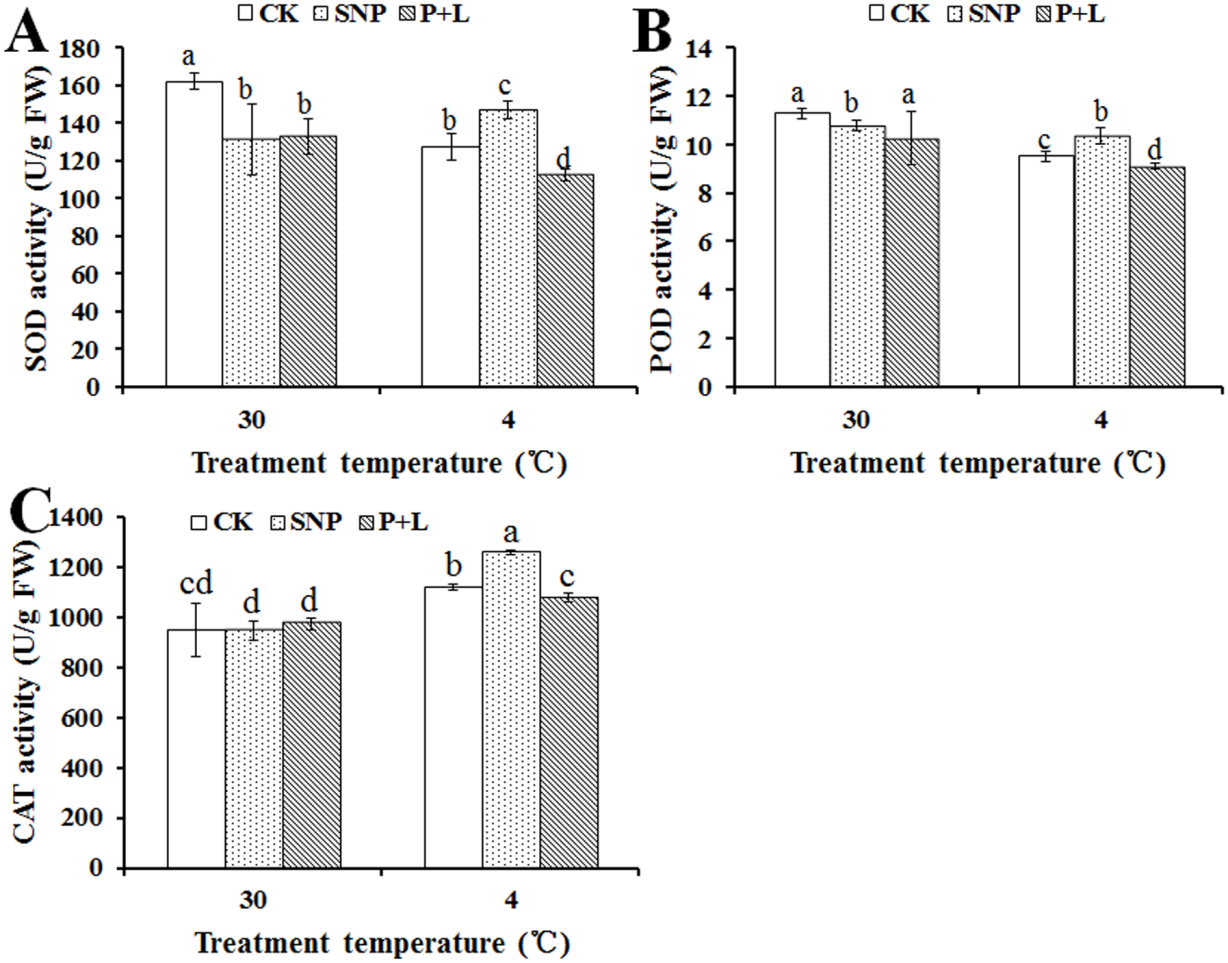
Alteration of antioxidant enzymes activities in the leaf of bermudagrass after treated with nitric oxide (NO) donor and inhibitor under cold stress. (A) Activities of superoxide dismutase (SOD); (B), Activities of peroxidase (POD), (C) Activities of catalase (CAT). Experiments were repeated for three times, and means were average values of activities of SOD, POD and CAT, respectively. Independent-samples *t* test was used to determine statistical differences. *Bars* show standard deviation. Different letters indicate statistical difference significance at *P* < 0.05 among the treatments. CK was control that treated with sterilized water; SNP was plant treated with SNP, P+L was plant treated with PTIO and L-NAME. FW was fresh weight.

Antioxidants could scavenge ROS induced by chilling stress, therefore the activity of antioxidant enzymes are markers of cold tolerance of the plant. Under chilling stress, SOD activity of the plant treated with SNP was 15.4% higher than that of control (127.56 U/g), while, SOD activity of the plant that treated with L-NAME plus PTIO was 11.9% lower than that of control (Fig 3A). Similar results were also observed in POD and CAT activities. Under chilling stress, POD and CAT activities of the plant treated with SNP were 8.8% and 12.5% higher than that of control (9.52 U/g), respectively. However, POD and CAT activities of the plant that treated with L-NAME plus PTIO were 4.3%, 3.8% lower than that of control, respectively (Fig 3B and 3C).

### Effects of SNP and L-NAME plus PTIO on OJIP transient curves and the JIP test of bermudagrass

**Fig 4:**
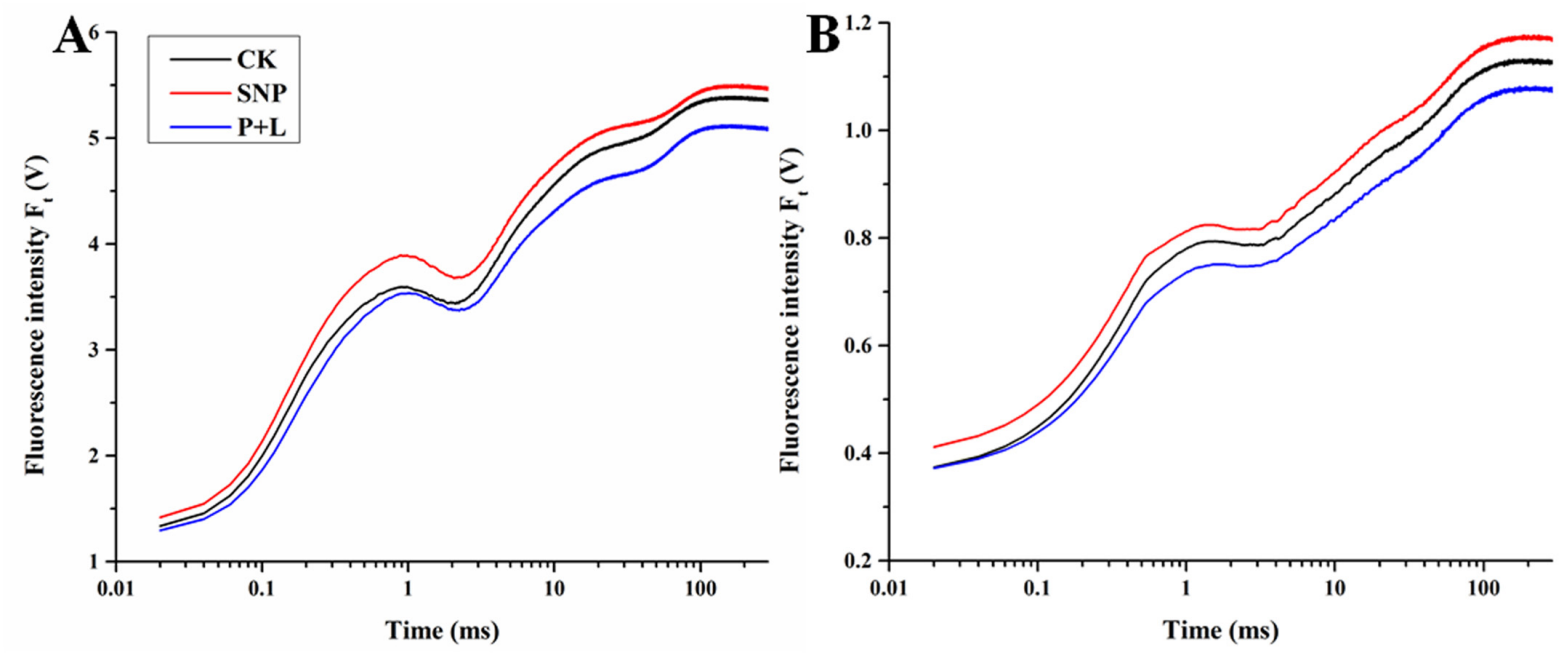
Alteration of chlorophyll fluorescence transients in bermudagrass leaves after treated with nitric oxide (NO) donor and inhibitor under cold stress. (A) Alteration of chlorophyll fluorescence transients after different treatments under normal temperature (30°C). (B) Alteration of chlorophyll fluorescence transients after different treatments under low temperature (4°C).

OJIP transient of bermudagrass was remarkably affected by low temperature. At 30°C, plant treated with SNP had the highest level of OJIP curves than that treated with L-NAME plus PTIO although it was not significant (Fig 4A). After the plants were treated with 4°C for 3 days, the OJIP curves dropped dramatically for all of the treatments. As a result, the plants treated with SNP the OJIP curves were higher than control, and plants that treated with L-NAME plus PTIO had a lower OJIP curves than control (Fig 4B).

The basic fluorescence parameters extracted from the OJIP curves were tabulated. The fluorescence recorded at 20 μs was regarded as *F*_*0*_. The results indicated that under cold stress, *F*_*0*_ of the plants treated with NO donor and inhibitor was higher and lower than that of control, respectively. At 30°C, *F*_*0*_ had no difference among different treatments. Meanwhile, this consequence was also found in *F*_*m*_, *F*_*J*_, *F*_*I*_ and *F*_*300μs*_ (known as K-step). Under cold stress, values of *F*_*m*_ and *F*_*I*_ of the plants that were treated with SNP were 8.3% and 2.2% higher than that of control, while, both of these parameters of the plants treated with L-NAME plus PTIO had no difference. *F*_*J*_ of the plants treated with SNP was 4.1% higher than that of control, while, it was 5.4% lower than that of control in the L-NAME plus PTIO treatment. Similarly, *F*_*300μs*_ of the plants treated with SNP was 3.4% higher than that of control, and it was 6.8% lower than that of control in the L-NAME plus PTIO treatment (Table 2).

**Table 2:** Basic photosynthetic parameters of the OJIP transient curves.

	*F*_*0*_	*F*_*m*_	*F*_*i*_	*F*_*j*_	*F*_*300μs*_
NT-CK	1.04±0.017a	4.58±0.1a	4.03±0.14a	3.16±0.09a	3.05±0.08a
NT-SNP	1.05±0.028a	4.63±0.08a	4.12±0.06a	3.21±0.07a	3.08±0.05a
NT-P+L	1.17±0.085a	4.57±0.11a	3.96±0.13a	3.19±0.13a	3.04±0.07a
LT-CK	0.51±0.02d	1.08±0.02b	0.85±0.03b	0.74±0.01d	0.59±0.01d
LT-SNP	0.54±0.01c	1.17±0.05c	0.94±0.04c	0.77±0.033c	0.61±0.015c
LT-P+L	0.48±0.01b	1.1±0.03b	0.9±0.01b	0.7±0.014b	0.55±0.01b

Values were listed as Mean ± SD. Independent-samples *t* test was used to determine statistical differences. Different letters indicate statistical difference significance at *P* < 0.05 among the treatments. NT means normal temperature (30°C), and LT means low temperature (4°C). CK was control that treated with sterilized water, SNP was plant treated with SNP, P+L was plant treated with PTIO and L-NAME.

**Fig 5:**
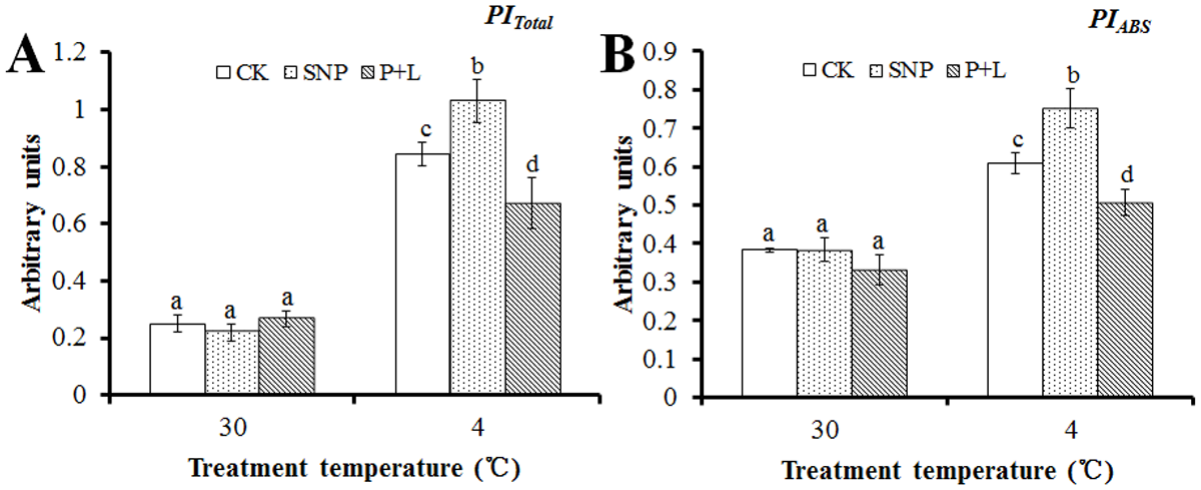
Alteration of performance index (PI) that deduced by JIP-test analysis of fluorescence transients. Calculations of each parameter refer to the method of Yusuf et al. (2010). (A) Alteration of PI for energy conservation from exciton to the reduction of PSI end acceptors (*PI*_*Total*_). (B) Alteration of PI for energy conservation from exciton to the reduction of intersystem electron (*PI*_*ABS*_). Experiments were repeated for three times, and means were average values of the parameters. Independent-samples *t* test was used to determine statistical differences. *Bars* show standard deviation. Different letters indicate statistical difference significance at *P* < 0.05 among the treatments. CK was control that treated with sterilized water, SNP was plant treated with SNP, P+L was plant treated with PTIO and L-NAME. 30 means the culture temperature of 30°C, and 4 means the culture temperature of 4°C.

**Fig 6:**
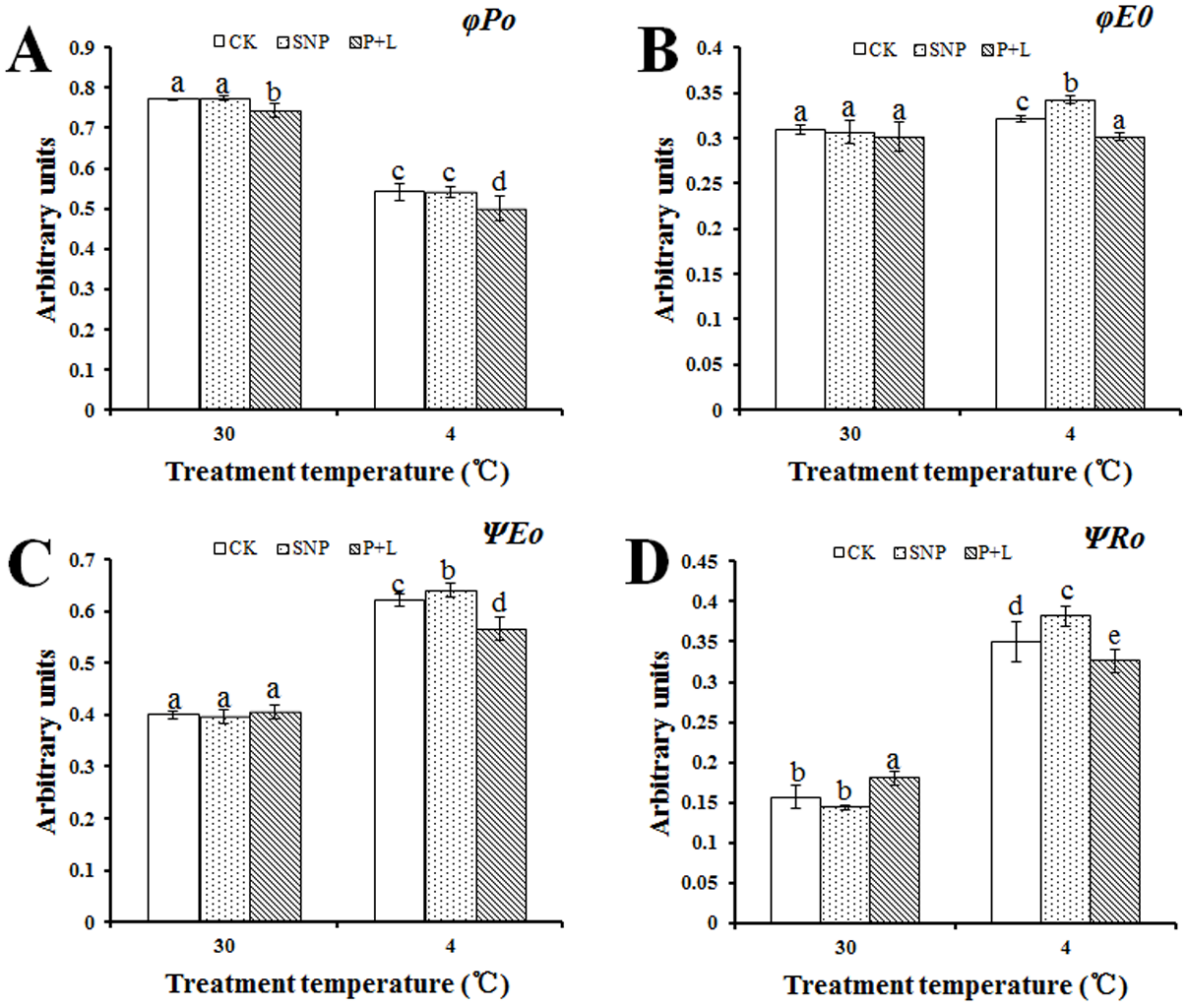
Alteration of quantum yields and efficiencies/probabilities that deduced by JIP-test analysis of fluorescence transients. Calculations of each parameter refer to the method of Yusuf et al. (2010). (A) Alteration of maximum quantum yield for primary photochemistry (*φP*_*0*_). (B) Alteration of quantum yield of the electron transport flux from Q_A_ to Q_B_ (*φE*_*0*_). (C) Alteration of efficiency/probability with that a PSII trapped electron is transferred from Q_A_ to Q_B_ (*ΨE*_*0*_). (D) Alteration of efficiency/probability with which an electron from Q_B_ is transferred until PSI acceptors (*ΨR*_*0*_). Experiments were repeated for three times, and means were average values of the parameters. Independent-samples *t* test was used to determine statistical differences. *Bars* show standard deviation. Different letters indicate statistical difference significance at *P* < 0.05 among the treatments. CK was control that treated with sterilized water; SNP was plant treated with SNP, P+L was plant treated with PTIO and L-NAME. 30 means the culture temperature of 30°C, and 4 means the culture temperature of 4°C.

**Fig 7:**
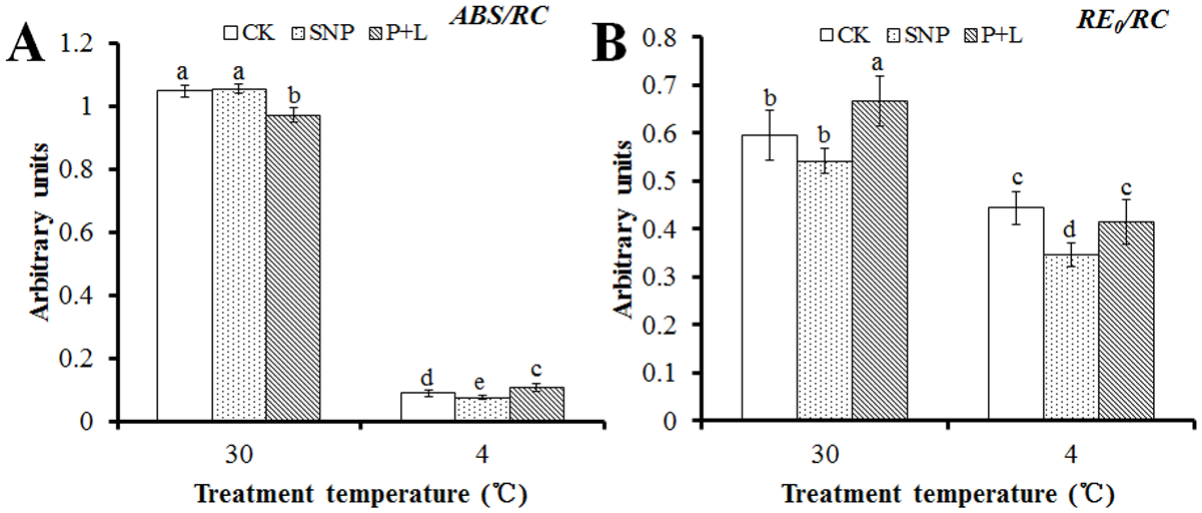
Alteration of energy fluxes per active PSII reaction center (RC) that deduced by JIP-test analysis of fluorescence transients. Calculations of each parameter refer to the method of Yusuf et al. (2010). (A) Alteration of absorbed photon flux per RC (*ABS/RC*). (B) Alteration of electron flux reducing end electron acceptors at PSI acceptor side per RC (*RE*_*0*_ */RC*). Experiments were repeated for three times, and means were average values of the parameters. Independent-samples *t* test was used to determine statistical differences. *Bars* show standard deviation. Different letters indicate statistical difference significance at *P* < 0.05 among the treatments. CK was control that treated with sterilized water, SNP was plant treated with SNP, P+L was plant treated with PTIO and L-NAME. 30 means the culture temperature of 30°C, and 4 means the culture temperature of 4°C.

The basic fluorescence parameters above were used in JIP-test to deduce structural and functional parameters for quantifying the photosynthesis in leaves. The results indicated that under cold stress, in the plants that were treated with SNP, the values of *φE*_*0*_, *ΨE*_*0*_, *ΨR*_*0*_, *PI*_*ABS*_ and *PI*_*Total*_ were higher than that of control. In detail, *PI*_*Total*_ and *PI*_*ABS*_ were 22.6% and 23% higher than that of control after treated with SNP, respectively. While, the values were 25.4% and 19.6% lower than that of control after L-NAME plus PTIO treatment, respectively (Fig 5A and 5B). Values of *φE*_*0*_, *ΨE*_*0*_, *ΨR*_*0*_ increased 6.3%, 3.2% and 8.6%, respectively as well. And as expected, in the plants that were treated with NO inhibitors, these values decreased 6.7%, 8.8% and 9.4%, respectively (Fig 6B–6D). Besides, the value of *φP*_*0*_ also decreased 8% in the plants that were treated with NO inhibitors (Fig 6A). However, unexpected, the values of *ABS/RC* and *RE*_*0*_ */RC* dropped in the plants that were treated with NO donor, which were 18.7% and 28.3%, respectively. But the value of *ABS/RC* increased 21.3% in the plants that were treated with NO inhibitors (Fig 7A and 7B).

### Effects of SNP and L-NAME plus PTIO on gene expression of bermudagrass

**Fig 8:**
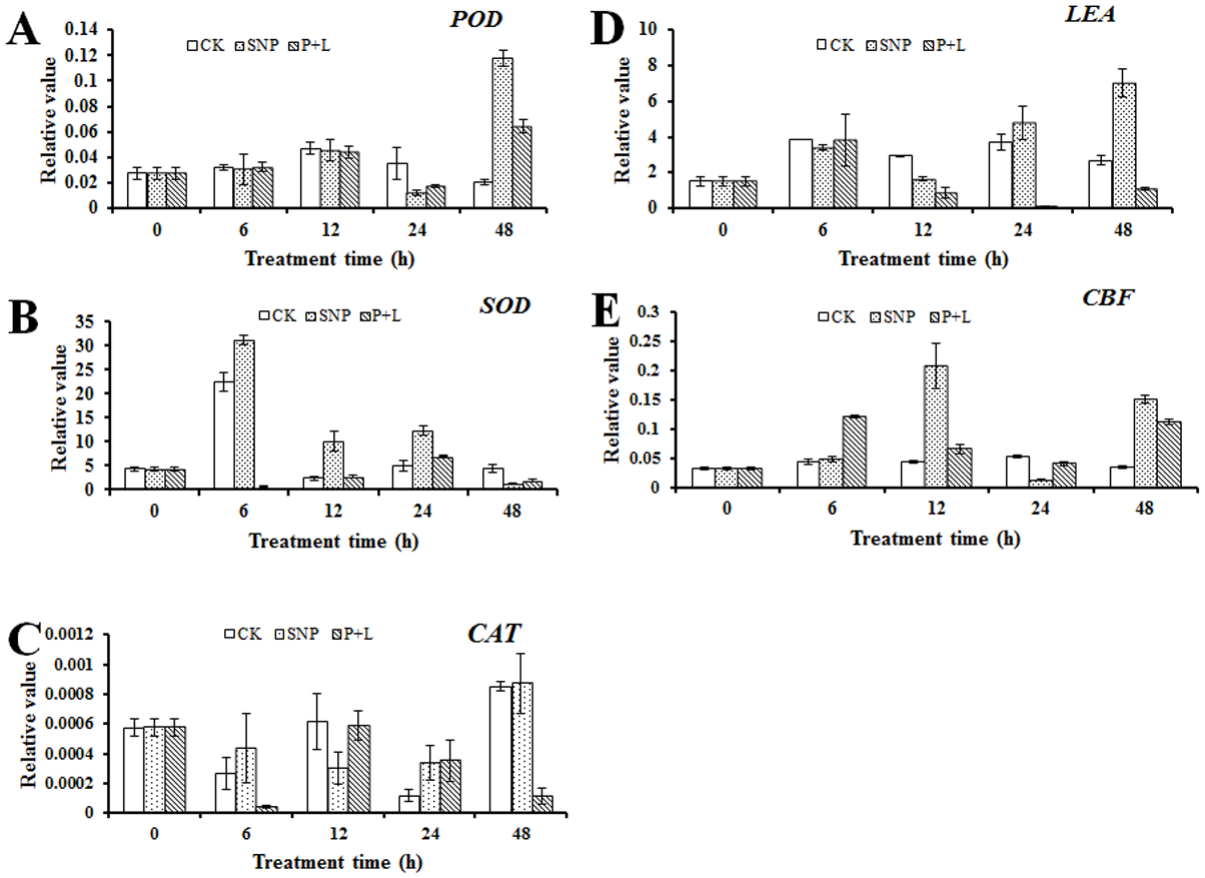
Expression pattern of genes in leaves of bermudagrass with different treatment under cold stress. (A) Expression of *POD*. (B) Expression of *SOD*. (C) Expression of *CAT*. (D) Expression of *LEA*. (E) Expression of *CBF*. Total RNA were isolated from leaves treated at 4°C for 6, 12, 24, and 48 h, respectively. Quantitative real time PCR was repeated for three times. Independent-samples *t* test was used to determine statistical differences. Bars show standard deviation.

To detect expression of the genes that related to cold stress, antioxidase genes *POD*, *SOD* (*Cu/Zn SOD*) and *CAT*, as well as *CBF* and *LEA* expression were measured by qRT-PCR. The results showed that expression of *POD* was highly up-regulated at hour 48 after treated with SNP under cold stress, although it was slightly upregulated after treated with PTIO and L-NAME (Fig 8A). *SOD* was dramatically up-regulated at hour 6 after treated with SNP under cold stress, even at hour 12 and 24 it was up-regulated significantly. While, expression of *SOD* was repressed after the plants were treated with NO inhibitors (Fig 8B). *CAT* was up-regulated at hour 48 after treated with SNP under cold stress, however, like *SOD*, expression of *CAT* was also repressed after treated with inhibitors (Fig 8C). *LEA* was up-regulated at hour 6, 24 and 48 after treated with SNP under cold stress, and it was repressed as expected after treated with inhibitors although it was slightly up-regulated at hour 6 (Fig 8D). *CBF* was dramatically up-regulated at hour 12 and 48 after treated with SNP under cold stress. However, it was up-regulated at hour 6 and hour 48 after treated with PTIO plus L-NAME, but the degree of up-regulation was significantly lower than that treated with SNP (Fig 8E).

## Discussion

Cold stress could induce lipid peroxidation and increase plasma membrane permeability of plant cells [7], and this phenomenon was also reported in bermudagrass [27]. In addition, cold stress could promote reactive oxygen species (ROS) formation in plants and lead to oxidative damage to plant. NO was reported to participate in counteracting the effects of ROS in multiple stressful conditions [28]. In this study, the results also indicated that NO was a critical factor involved in cold resistance of bermudagrass.

The MDA content and relative EL value of leaves were lower in the plants which were treated with SNP, but higher in the plant that were treated with PTIO plus L-NAME, as compared to controls (Fig 1A and 1B). The results implied that exogenous applied NO could protect the cell membrane against damage imposed by cold stress, whereas, in the absence of NO, the cell membrane stability was more severely damaged.

The antioxidant enzymes function as ROS scavengers. The activities of SOD, POD and CAT were found to be increased in response to various oxidative stresses [9,29]. The main functions of antioxidant enzymes were to eliminate O_2_^-^ radicals to prevent the oxidation of biological molecules. It was also reported that NO was an O_2_^-^ scavenger because it could react with O_2_^-^ to form NO_2_^-^ and NO_3_^-^ [30]. In transgenic tobacco (*Nicotiana tabacum*) it was reported that NO was involved in suppressing of ROS accumulation [31]. The results showed that activities of SOD, POD and CAT increased in plants treated with SNP than those of controls. By contrast, the activities of the antioxidant enzymes were lower after treatment with L-NAME plus PTIO than controls (Fig 3A–3C). The findings suggested that NO could improve cold resistance of bermudagrass through increasing the antioxidant enzymes activities. In addition, a slight difference in SOD and POD activities were detected in the different treated-plants under 30°C condition. These results may due to exogenous NO changed the endogenous NO signal pathway, thereafter affected the SOD and POD activities under 30°C condition. POD activity decreased after NO treatment was also reported by Kopyra and Gwóźdź [32]. However, why SOD activities were not reverted by PTIO+L-NAME remains unclearly.

Change of the antioxidant enzymes activities may be due to the alteration of expression of the corresponding genes. Positive correlation between antioxidase (such as SOD, POD and APX) and gene expression was reported in perennial ryegrass under salinity and lead stress [24,29]. Similar results were detected in bermudagrass under cold stress [33]. The results of this study showed that under cold stress, after treated with SNP, expression of the antioxidase genes such as *POD*, *SOD* and *CAT* were up-regulated dramatically. While after plants were treated with PTIO plus L-NAME, expressions of the genes were up-regulated slightly even been repressed (Fig 8A–8C). These results were consistent with the conclusion that there was positive correlation between enzymes activities and genes expression. *CBFs* were typical genes that related to cold stress in plants [34]. *LEA* genes were also reported to involve in cold acclimation [3]. As it was shown in the results, *CBF* and *LEA* were up-regulated after treated with SNP under cold stress, while expression of genes were repressed after treated with NO inhibitors (Fig 8D and 8E). These results implied NO indeed affected cold resistance of bermudagrass through influencing the expression of the correlated genes.

According to previous study, photosynthetic sites were endogenous NO cellular source [35]. As well as exogenous NO should also may affect the reaction of photosystem in plant, so the fluorescence curve was detected in this study. The OJIP fluorescence transient includes abundant information that can be used to calculate the parameters by JIP-test which quantifies the flow of energy through PSII at the level of reaction center (RC) [36]. *F*_*0*_ stands for the fluorescence emission after dark adaption for all of the primary quinine acceptors (Q_A_) were oxidized. Precisely, higher *F*_*0*_ value indicated higher physical separation between the PSII reaction center and the associated pigment antennae [37]. In the plants that were treated with NO donor, *F*_*0*_ value was higher than that of control. While, after PTIO plus L-NAME treatment, it was lower than that of control (Table 2), this suggested that NO was essential in cold resistance in bermudagrass. *φP*_*0*_ suggested the quantum yield of excitation energy that was trapped by PSII antenna pigments, it is equivalent to the efficiency of the light reactions (*F*_*v*_ */F*_*m*_), and this fluorescence ratio is widely used to estimate the damage to PSII activity [11]. It was reported that, under chilling temperature *F*_*v*_ */F*_*m*_ decreased drastically in plant [38]. *φE*_*0*_ was the quantum yield for electron transport, and *ΨE*_*0*_ was the probability that a trapped exciton moves an electron into the electron transport chain beyond Q_A_^-^. Meanwhile, the index *PI*_*total*_ represented the overall behavior of the photosynthetic activities; this parameter combines the three primary functional steps, light energy absorption step, excitation energy trapping step, and conversion of excitation energy to electron transport step, of photosynthetic activity through a reaction center complex of PSII into a single multi-parametric expression [39]. According to the results obtained under cold stress, after SNP treatment these parameters were higher and lower after L-NAME plus PTIO treatment than those of control, respectively (Figs 5 and 6). Increase/decrease of *ABS/RC* ratio reflects increase/decrease in the number of the active RC per chlorophyll concentration. *PI*_*ABS*_ is the density of RC that is expressed per absorption. *ET*_*0*_ */RC* suggests the electron transport in the active reaction center. These indexes were also found changed in the results (Fig 7). This implied that NO participated in electron transfer of PSII, and the photosynthetic reaction was sensitive to NO treatment under cold stress in bermudagrass.

## Conclusion

The role of NO on bermudagrass leaves against cold stress was investigated in this study. Under cold stress, stability of cell membrane was improved, activities of antioxidant enzyme and photosynthetic activity increased in bermudagrass after treated with exogenous NO. While, negative effects were found in these index after NO inhibitors treatment. These results suggest NO play a positive role in cold resistance of bermudagrass.
